# A Preliminary Metabolomic Study of Yorkshire Terrier Enteropathy

**DOI:** 10.3390/metabo12030264

**Published:** 2022-03-19

**Authors:** Alexandra I. Galler, Kristaps Klavins, Iwan A. Burgener

**Affiliations:** 1 Division of Small Animal Internal Medicine, University of Veterinary Medicine, 1210 Vienna, Austria; iwan.burgener@vetmeduni.ac.at; 2 Rudolfs Cimdins Riga Biomaterials Innovations and Development Centre of RTU, Institute of General Chemical Engineering, Faculty of Materials Science and Applied Chemistry, Riga Technical University, Pulka St 3, LV-1007 Riga, Latvia; kristaps.klavins_3@rtu.lv; 3 Baltic Biomaterials Centre of Excellence, Headquarters at Riga Technical University, Pulka St 3, LV-1007 Riga, Latvia

**Keywords:** inflammatory bowel disease, chronic inflammatory enteropathy, protein-losing enteropathy, bile acids, acylcarnitines, fatty acids

## Abstract

Perturbations of metabolite profiles in human and canine enteropathies have been reported before. However, data in dogs are scarce and inconsistent. Currently, the metabolite profile in Yorkshire Terrier enteropathy (YTE) and the impact of treatment is unknown. The objective of this study was to investigate the plasma metabolome of 13 Yorkshire Terriers with YTE and compare it to 20 healthy Yorkshire Terriers. Furthermore, we studied the impact of treatment on the metabolome. In this prospective observational study, plasma metabolite profiles were analyzed by flow injection analysis-tandem mass spectrometry (FIA-MS/MS) and liquid chromatography-tandem mass spectrometry (LC-MS/MS) using a targeted metabolomics kit. Metabolite analysis revealed that YTE is accompanied by changes in lipid and bile acid metabolism. YTE was associated with a significant decrease of long-chain fatty acids (octadecenoic acid, eicosadienoic acid, eicosatrienoic acid) and lower levels of long-chain acylcarnitines (tetradecanoylcarnitine, hexadecanoylcarnitine, hexadecenoylcarnitine, octadecenoylcarnitine) compared with healthy controls. Furthermore, taurodeoxycholic acid, a secondary bile acid, was decreased in plasma from YTE patients. These changes might be breed-specific and might be involved in the pathogenesis of YTE. Interestingly, changes in metabolite levels were not recovered after treatment and differed considerably from healthy controls.

## 1. Introduction

Inflammatory bowel disease (IBD) is a common cause of chronic or relapsing gastrointestinal (GI) signs in dogs. Canine IBD is diagnosed by excluding systemic, endocrine, neoplastic, and infectious causes for GI signs and histologic evidence of intestinal mucosal inflammation [1,2]. The etiology of IBD is not well understood. However, the disease is multifactorial, arising from a complex interplay between the genetic background, environmental factors, the intestinal innate and adaptive immune system, and the intestinal microbiome [3,4,5,6]. Due to its complexity, mechanistic understanding of the disease is limited, and research has relied primarily on mouse models in which GI inflammation is induced by various experimental methods [7]. Metabolomic phenotyping provides new mechanistic insights and could potentially address key issues of IBD and provide valuable tools in clinical diagnosis and assessment of treatment response.

Several studies on canine enteropathy have reported changes in metabolite profiles, especially in the short-chain fatty acid and amino acid metabolism; however, data are scarce and inconsistent [8,9,10,11,12,13]. Confounding factors like previous treatment, breed, age, sex, and different biological samples and methodological approaches may have caused discrepancies between previous studies [14]. Moreover, discordant results may be caused by the multifactorial nature of IBD and the high variation in phenotypes and severity. IBD may resemble a syndrome comprising several disease subtypes. More than 240 risk loci have been associated with adult-onset IBD in humans [15]. On the other hand, very early-onset IBD developing in children under 6 years has been associated with approximately 50 genes causing monogenic forms of IBD, representing more homogeneous subtypes of the disease [16]. Less is known about IBD subtypes in dogs. Although IBD can affect any dog, some breeds display breed-specific disease phenotypes [17,18,19]. In Yorkshire Terriers, retrospective studies have described an enteropathy that is distinct from other breeds [17,18], suggesting the existence of a breed-specific “Yorkshire Terrier enteropathy” (YTE). In addition to the classic GI signs, Yorkshire Terriers often show clinical signs associated with hypoalbuminemia and low oncotic pressure. While intestinal lymphoplasmacellular infiltration and lymphangiectasia have been reported in many dogs with IBD [2], severe intestinal lymphatic dilation, crypt lesions, and villous stunting are consistent histopathologic findings in YTE [18]. Inbreeding in modern dog breeds is comparable to geographically highly isolated human populations, thus offering an ideal population structure for exploring disease mechanisms of various complex disorders, including IBD. The metabolome of YTE has not been investigated so far and may differ considerably from the metabolome in IBD dogs of various other breeds. Furthermore, limited information is available concerning the influence of different treatments on the metabolome in canine IBD.

The objective of this study was to investigate the plasma metabolome of treatment naïve Yorkshire Terriers with YTE and compare it to healthy Yorkshire Terriers. Furthermore, we studied the impact of treatment on metabolite profiles. For this, we relied on a standardized assay that enables a targeted metabolomics analysis with a broad range of metabolites and relevant pathways. In this study, we hypothesized that YTE would be associated with changes in plasma metabolite levels and that the plasma metabolite profile of dogs in clinical remission would be different from dogs with an active disease resembling more closely the profile of healthy dogs.

## 2. Results

### 2.1. Animal Characteristics

The YTE group (*n* = 13) included 9 females (4 spayed) and 4 males (2 neutered) Yorkshire Terriers. The mean age was 6.4 years (+/−1.7 years), and the mean body weight was 3.7 kg (+/−1.5 kg). At the time of presentation, dogs were fed various commercial diets (12 canned and 1 dry food). The primary reasons for the presentation were a history of chronic or intermittent GI signs in 10 dogs followed by pleural and/or abdominal effusion in 3 dogs. Mean fecal scores were 4.5 out of 7 (+/−1.1), and body conditioning scores were 4.3 out of 9 (+/−1). Muscle conditioning scores were A (from A to D) in 9 dogs, B in one, C in two, and D in one dog. The dogs were suffering from mild to severe YTE (mean CCECAI score of 9.2 +/− 3). Duodenal WSAVA scores indicated mild or moderate histological changes (median 3.5, range 2–11). In all dogs, a predominantly lymphocytic-plasmacytic infiltration of the intestinal mucosa was found. Other pathohistological abnormalities were villus blunting (*n* = 4), crypt lesions (*n* = 5), lymphangiectasia (*n* = 3) and additional eosinophilic (*n* = 4) or neutrophilic (*n* = 3) inflammatory infiltration.

Ten dogs were available for re-evaluation after reaching clinical remission. One dog was still in the treatment trial at the sample analysis time. One dog had to be excluded due to poor owner compliance, and another dog had to be excluded due to poor acceptance of the offered diets. From the 10 re-evaluated dogs, 8 were clinically well-controlled, received a hydrolyzed diet, and were resampled 69–86 days (median 74 days) post-diagnosis. Two dogs reached clinical remission only after prednisolone administration and were resampled 126 and 134 days post-diagnosis (71 respectively 74 days after initiation of prednisolone). Fecal scores (mean: 2 +/− 0.5), body conditioning scores (mean: 4.7 +/− 0.7) and muscle conditioning scores (A, *n* = 7; B, *n* = 1; C = 1) had improved in all dogs.

The control group (*n* = 20) consisted of 10 females (6 spayed) and 10 males (5 neutered) Yorkshire Terriers. The mean age of the dogs was 8.2 years (+/−3.2 years). The mean body weight was 3.9 (+/−1.3kg). Nineteen dogs in the control group received various commercial diets (14 canned and 5 dry food). One dog was fed with a Bones and Raw Food (BARF) diet. Mean fecal scores were 2 (+/−0.8), mean body conditioning scores 5.5 (+/−0.8) and muscle conditioning scores were A (*n* = 15) or B (*n* = 5). The median CECCAI score in the healthy control group was 0.5 (range 0–2). Neither age nor sex or bodyweight was significantly different between dogs with YTE and healthy controls.

### 2.2. Metabolomics

The used metabolomics kit potentially measures 630 metabolites, however, 108 had to be excluded from statistical analysis because they were below the limit of detection in 20% or more of subjects. After data cleaning, 522 metabolites remained for statistical analysis, including amine oxides (1), amino acids (20), amino acid-related metabolites (23), bile acids (6), biogenic amines (5), carbohydrates and related (1), carboxylic acids (2), cresols (1), fatty acids (7), hormones (1), indoles and derivatives (3), nucleobases and related (2), vitamins and cofactors (1), acylcarnitines (31), phosphatidylcholines (74), lysophosphatidylcholines (14), sphingomyelins (15), ceramides (23), dihydroceramide (2), hexosylceramides (13), dihexosylceramides (9), trihexosylceramides (6), cholesteryl esters (20), diglycerides (14) and triglycerides (228).

### 2.3. Data Statistical Analysis

#### 2.3.1. Multivariate Analysis

**Figure 1:**
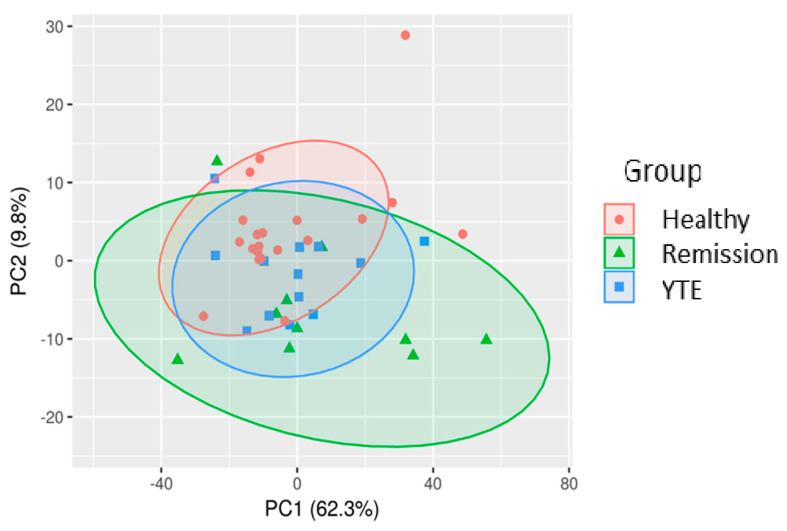
Principal component analysis (PCA) score plot of the first principal component (PC1) and second principal component (PC2) of plasma metabolite data from healthy controls, dogs with YTE, and dogs with YTE in clinical remission. The amount of variance in percent accounted for by each PC is included in brackets. Ellipses represent the 95% confidence interval of metabolite profiles for each group.

The principal component analysis (PCA) showed no clear separation between groups. Plasma metabolite profiles of YTE dogs showed a considerable variation, while metabolite profiles of the healthy control clustered more tightly (Figure 1).

#### 2.3.2. Univariate Analysis

##### YTE Versus Healthy Dogs

**Figure 2:**
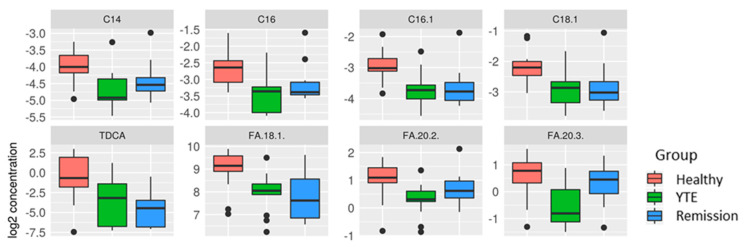
Box plots comparing metabolites after log-2 transformation in healthy controls, dogs with YTE, and dogs with YTE in clinical remission. Boxplots of metabolites with significant differences between healthy dogs and YTE dogs are shown. Box plots represent the 25–75th percentile; the median is shown as the heavy dark horizontal line, and vertical lines extend to the minimum and maximum values. Dots represent outliers. Legend: C14, tetradecanoylcarnitine; C16, hexadecanoylcarnitine; C16:1, hexadecenoylcarnitine; C18:1, octadecenoylcarnitine; TDCA, taurodeoxycholic acid; FA18:1, octadecenoic acid; FA 20.1; eicosadienoic acid; FA 20:3, eicosatrienoic acid; YTE, Yorkshire Terrier enteropathy.

First, metabolite profiles of YTE were compared with healthy controls. Out of 522 metabolites and lipids analyzed, 8 showed significantly altered plasma levels in YTE dogs. Plasma concentrations of 4 long-chain-acylcarnitines; tetradecanoylcarnitine (*p* < 0.001, q = 0.063), hexadecanoylcarnitine (*p* < 0.001, q = 0.062), hexadecenoylcarnitine (*p* < 0.001, q = 0.063) and octadecenoylcarnitine (*p* = 0.002, q = 0.132) were decreased in dogs with YTE. Three unsaturated long-chain-fatty acids; octadecenoic acid (*p* < 0.001, q = 0.132), eicosadienoic acid (*p* = 0.002, q = 0.132) and eicosatrienoic acid (*p* < 0.001, q = 0.062) were decreased in YTE plasma samples compared to controls (Figure 2). Seven primary bile acids and 7 secondary bile acids were measured. Glycine conjugated bile acids (n = 6) were below the limit of detection, which is due to a species-specific lack of glycine conjugated bile acids in dogs [20]. TDCA, a secondary bile acid was decreased in YTE (*p* = 0.002, q = 0.162). (Figure 2).

**Figure 3:**
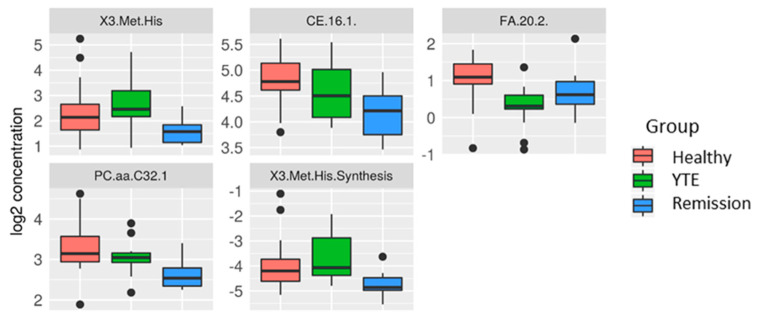
Box plots comparing metabolites after log-2 transformation in healthy controls, dogs with YTE, and dogs with YTE in clinical remission. Box Plots of metabolites with differences between clinically active YTE and YTE dogs in remission are shown. Box plots represent the 25–75th percentile; the median is shown as the heavy dark horizontal line. Vertical lines extend to the minimum and maximum values. Dots represent outliers. Legend: X3. Met. His, 3-Methionine-histidine; CE, ceramide; FA 20:2, eicosadienoic acid; PC, Phosphatidylcholine; YTE, Yorkshire Terrier enteropathy.

Active YTE versus remission. Plasma metabolite profile response to the effects of medical treatment was also investigated. Although we saw changes in the metabolite levels of dogs in clinical remission compared with untreated dogs, significance was lost after correction for multiple testing. Metabolites reaching significance levels of *p* < 0.01 in statistical analysis were eicosadienoic acid (*p* = 0.009, q = 0.795) which increased in YTE dogs after treatment, 3-Methionine-histidine (*p* = 0.007, q = 0.795) as well as a ceramide (*p* = 0.003, q = 0.795) and a phosphatidylcholine compound (*p* = 0.003, q = 0.795) which were decreased in treated dogs (Figure 3). Bile acids, long-chain-acylcarnitines, and long-chain fatty acids were still reduced. (Figure 2).

##### YTE Remission Versus Healthy Controls

**Figure 4:**
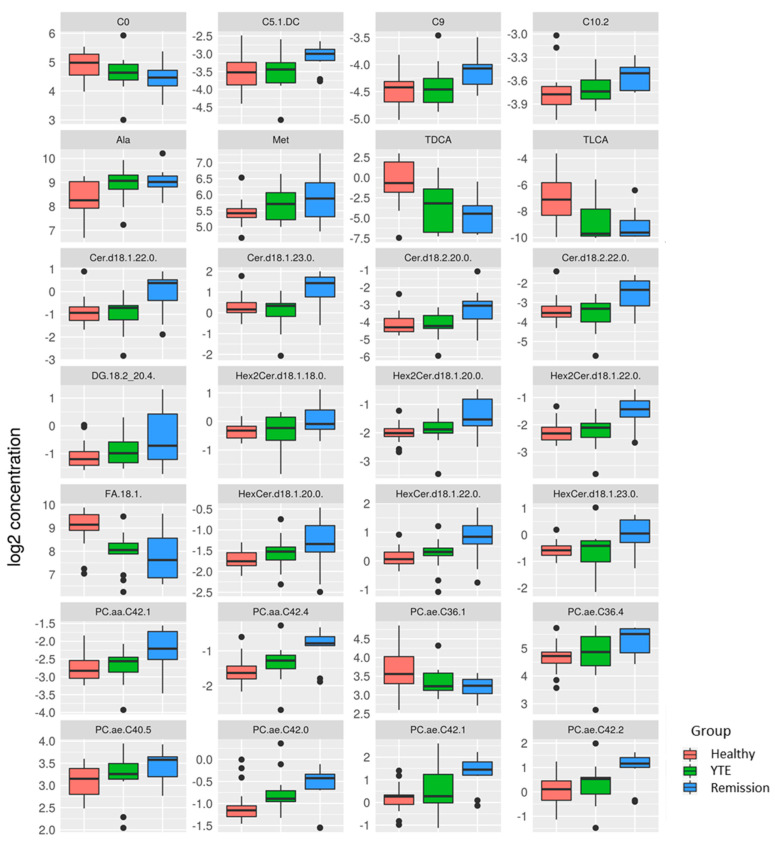
Box plots comparing metabolites after log-2 transformation in healthy controls, dogs with YTE, and dogs with YTE in clinical remission. Box plots of metabolites with significant differences between healthy dogs and YTE dogs in remission are shown. Box plots represent the 25–75th percentile; the median is shown as the heavy dark horizontal line. Vertical lines extend to the minimum and maximum values. Dots represent outliers. Legend: C, carnitine; Ala, alanine; Met, methionine; TDCA, taurodeoxycholic acid; TLCA, taurolithocholic acid; Cer.d, ceramide; DG, diaglyceride; Hex2Cer.d, dihexosylceramide; FA, fatty acid; HexCer.d, hexosylceramide; PC, phosphatidylcholine.

**Figure 5:**
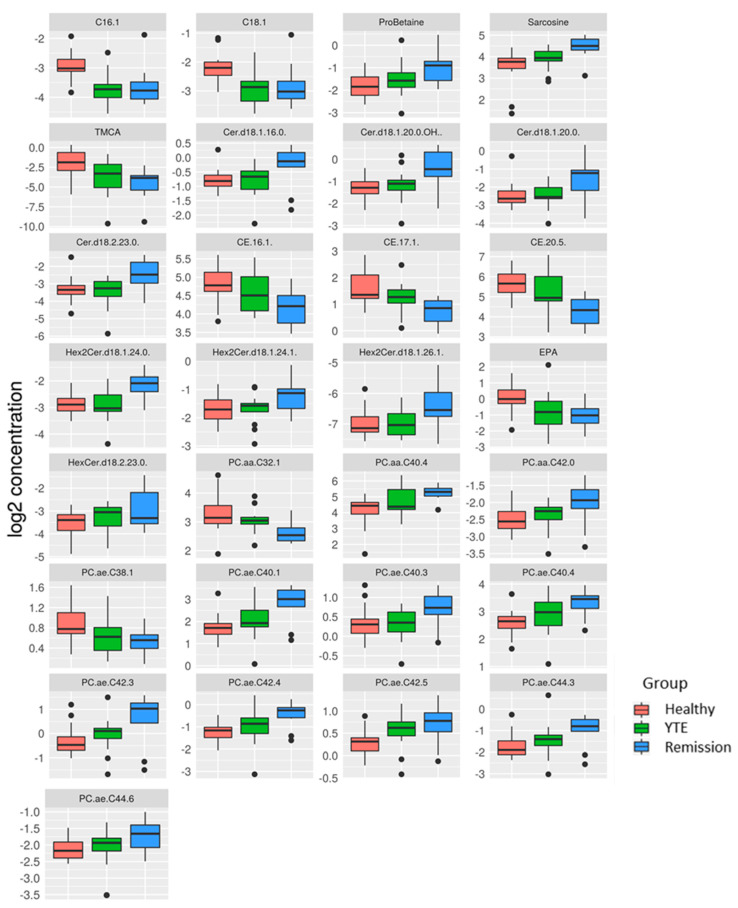
Box plots comparing metabolites after log-2 transformation in healthy controls, dogs with YTE, and dogs with YTE in clinical remission. Box plots of metabolites with significant differences between healthy dogs and YTE dogs in remission are shown. Box plots represent the 25–75th percentile; the median is shown as the heavy dark horizontal line. Vertical lines extend to the minimum and maximum values. Dots represent outliers. Legend: C, carnitine; TMCA, tauro-muricholic acid; Cer.d, ceramide; CE, cholesteryl ester; Hex2Cer.d, dihexosylceramide; EPA, eicosapentaenoic acid; HexCer.d, hexosylceramide; PC, phosphatidylcholine.

**Figure 6:**
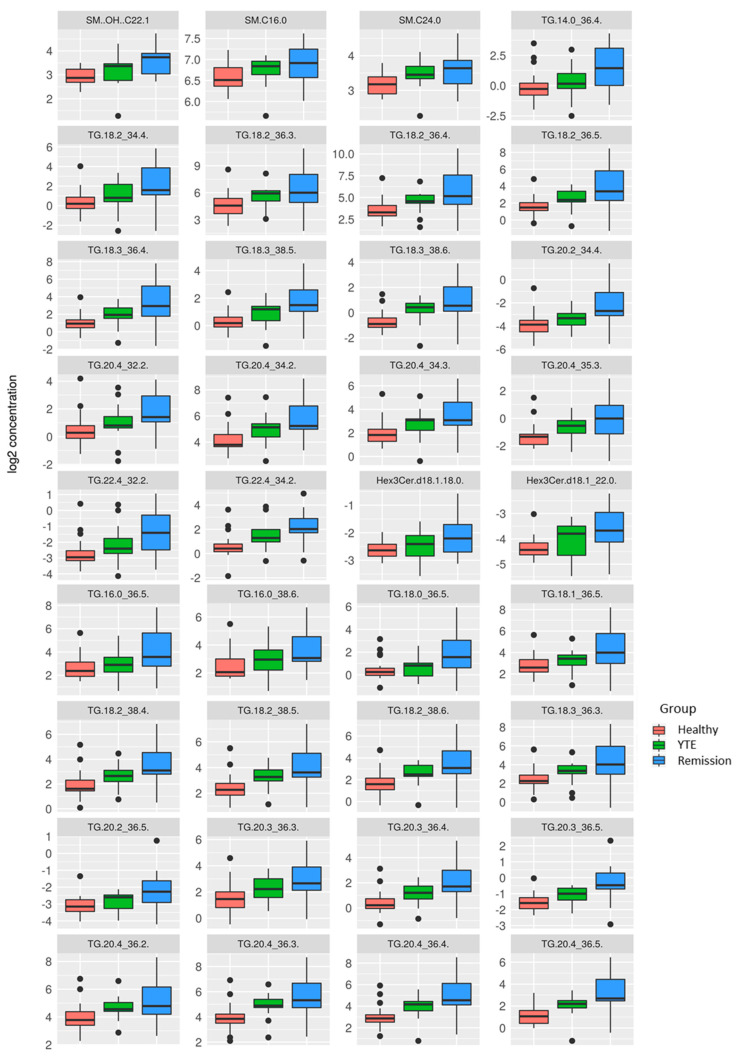
Box plots comparing metabolites after log-2 transformation in healthy controls, dogs with YTE, and dogs with YTE in clinical remission. Box plots of metabolites with significant differences between healthy dogs and YTE dogs in remission are shown. Box plots represent the 25–75th percentile; the median is shown as the heavy dark horizontal line. Vertical lines extend to the minimum and maximum values. Dots represent outliers. Legend: SM..OH.., hydroxysphingomyelin; SM, sphingomyelin; TG, triaglyceride; Hex3cer.d, trihexosylceramide.

Dogs with clinically silent YTE were compared to healthy dogs, and the most significant differences were detected in this group comparison. 93 metabolites were significantly changed. Dogs in clinical remission displayed markedly increased plasma levels of many lipids of different ontology classes as well as some polar metabolites, while bile acids and long-chain-acylcarnitines were lower in YTE dogs in clinical remission. (Figure 4, Figure 5 and Figure 6).

## 3. Discussion

Metabolomics provides the analysis of small molecules in biological samples. It is a powerful tool to assess metabolic changes associated with several pathological conditions. It is increasingly used to discover etiological factors, disease signatures and as a screening tool for different pathological conditions, including IBD [21,22]. Characterization of metabolic changes occurring in canine chronic enteropathies can increase our understanding of disease pathophysiology, improving diagnosis, treatment, and disease management.

In the current study, a quantitative targeted metabolomics approach was employed to identify changes in plasma metabolite levels in YTE before and after treatment. We detected lipid and bile acid metabolism changes in Yorkshire Terriers with YTE compared to healthy dogs. These changes were not recovered after treatment. We did not observe abnormalities in amino acids and related compounds (esp. tryptophan), carbohydrates, hormones, or vitamins in dogs with YTE. The most significantly altered metabolites belonged to the lipid group. Lipids are a heterogeneous group of hydrophobic and amphiphilic molecules that possess many functions, including maintaining membrane structure and permeability, trafficking and signal transduction, regulation of gene expression, and modulation of immunity and inflammation. Furthermore, lipids can alter tight junction permeability, control autophagy, and serve as antioxidants [23]. Alterations in lipid profiles and lipid homeostasis have been reported in human IBD [24,25,26,27,28,29]. The first published metabolomics study on canine IBD showed no significant alterations in lipid metabolism in IBD dogs compared to healthy controls [12]. A recent study revealed variances in the phospholipid blood profiles of dogs with steroid-responsive versus food responsive chronic enteropathy [30]. The lack of a healthy control group was a limitation of the study. Furthermore, only the phospholipid profile was assessed, while our study also investigated many other lipid classes. The current study revealed a significant depletion of long-chain free fatty acids in dogs with YTE, indicating lipid malabsorption or dysmetabolism. Depletion of free fatty acids in dogs with YTE may be caused by decreased intake caused by diminished appetite, vomiting, diarrhea, or malabsorption due to disturbed epithelial transport, reduced intestinal absorptive area, and/or accelerated intestinal transit time [31,32]. Moreover, YTE is often associated with dysfunctional intestinal lymphatics [17,18], which may cause breed-specific alterations in lipid absorption. However, YTE resembles a disease phenotype with widespread mucosal involvement. Therefore, malabsorption should affect a wide variety of macronutrients and micronutrients, not observed in the present study. A decrease in long-chain fatty acids could also have been caused by fatty acid hypermetabolism. In YTE, high energy demand for the recruitment of immune cells to the inflamed intestinal mucosa and mucosal repair may lead to a depletion of fatty acids. Alternatively, the lower amount of long-chain fatty acids may indicate altered lipid cascades in response to inflammation and immune regulation. Long-chain fatty acids have dual actions on intestinal inflammation. Oleic acid, eicosadienoic acid, and eicosatrienoic acid seem to play a role in modulating inflammatory responses. They have been demonstrated to possess anti-inflammatory properties by inhibiting various aspects of inflammation, including leukocyte chemotaxis, adhesion molecule expression, leukocyte-endothelial adhesive interactions, production of pro-inflammatory cytokines, and T cell reactivity [33,34,35,36]. Therefore, as observed in our study, a lack of these anti-inflammatory fatty acids may predispose affected dogs for an overshooting intestinal inflammatory response in the course of YTE. Alternatively, depletion may have occurred due to a higher demand for these anti-inflammatory lipids. Acylcarnitines are essential for transporting long-chain fatty acids through the mitochondrial membrane for beta-oxidation [37]. They are produced by conjugation of carnitine to a fatty acid, and their plasma concentration can be used as an indicator for the cellular metabolism of fatty acids, β-oxidation, and energy status [37,38]. In the present study, a significant decrease was found in the levels of several long-chain acylcarnitines in YTE, while carnitine and short-chain acylcarnitines were not significantly different between YTE and controls. The pattern of the carnitine ester profile found in our patients differs from that reported by previous studies. A study in humans with clinically asymptomatic celiac disease revealed lower plasma concentrations of short- and long-chain carnitine esters with unchanged carnitine levels compared to healthy controls [39]. In contrast, lower levels of short-chain acylcarnitines and carnitine but higher levels of long-chain acylcarnitines were detected in patients with ulcerative colitis compared to healthy controls [40]. A study investigating the serum metabolome in IBD dogs revealed elevated plasma-free carnitine and total acylcarnitine levels but decreased short-chain acylcarnitines compared to healthy control dogs [13]. The reason for these inconsistent results is not clear. It has been shown that intestinal inflammation causes mitochondrial dysfunction and disturbed beta-oxidation, but this should lead to higher concentrations of acylcarnitines, free carnitine, and fatty acids [41]. Intestinal dysbiosis may directly influence acylcarnitines since they can be metabolized by intestinal bacteria [42] and this could be a reason for the alterations observed in the present study. Furthermore, a lack of short-chain fatty acids leads to the increased consumption of luminally delivered long-chain acylcarnitines by intestinal epithelial cells [41], which could lead to a depletion of this fraction, as seen in the present study. Decreased fecal short-chain fatty acid concentrations have been shown to occur in dogs with chronic enteropathy [43]. Alternatively, considering the concomitant depletion of long-chain fatty acids in our study, malabsorption and disturbed metabolism of long-chain fatty acids could explain the acylcarnitine changes observed in the present study.

We have detected a decrease in taurodeoxychlic acid (TDCA), a conjugated secondary bile acid (BA), while other BAs were not altered in the plasma of dogs with YTE. BA malabsorption has been reported in a substantial proportion of patients with Crohn’s disease and with diarrhea-predominant irritable bowel syndrome in humans [44]. Furthermore, decreased expression of the active sodium-dependent BA transporter in the ileum of dogs with chronic enteropathy has been documented [45]. Therefore, one might expect decreased plasma levels of various BAs in dogs with YTE. However, with increased fecal BA loss, the liver increases BA synthesis to compensate for it [46]. 7α-hydroxy-4-cholesterin-3-one (C4), a BA precursor, can be measured in blood and used as an indicator of hepatic BA synthesis [47]. In a previous study, C4 was increased in a proportion of dogs with chronic diarrhea, indicating a compensatory increase of bile acid synthesis suggesting BA malabsorption in 3/17 dogs [48]. In humans, various methods can be used to diagnose bile acid malabsorption, including determining fecal bile acids, measuring retention of labeled bile acid analogs, and investigating plasma metabolites from bile acid synthesis [49]. These methods could be better suited for the assessment of bile acid malabsorption. However, dysbiosis in chronic enteropathies might influence the balance between primary and secondary bile acids. A small percentage of the intestinal luminal bile acids are not reabsorbed and reach the colon in physiological states. Colonic bacteria produce secondary bile acids by deconjugation and dehydroxylation of primary bile acids. These secondary bile acids can be reabsorbed and conjugated in the liver [50]. Clostridium hiranonis is a bacterial species that plays an essential role in converting primary to secondary BAs [51]. Decreased percentages of Clostridium hiranonis have been reported in dogs with chronic enteropathies [52,53]. Furthermore, dogs with chronic enteropathies had lower abundances of fecal secondary BAs compared to healthy dogs [52,54]. Therefore, dysbiosis and decreased production of secondary BAs could have caused decreased plasma levels of TDCA encountered in this study.

Amino acids have various functions and are essential as energy substrates for enterocytes, gastrointestinal growth, wound healing, maintenance of mucosal barrier function, and play a role in the intestinal immune system and oxidative stress [55,56]. In humans, serum amino acid profiles are used as non-invasive, predictive markers of intestinal inflammation [21], and amino acid supplementation can improve inflammatory changes and clinical signs in experimental animal models of colitis [57,58]. We did not detect differences in the plasma amino acid profile in YTE compared to healthy controls. This was surprising since previous studies revealed various amino acid alterations in dogs with chronic enteropathies [8,9,10,11,12,13]. The most constant findings in previous studies were low blood levels of tryptophan in dogs with protein-losing enteropathy [8] and IBD [9,10]. Our results might be specific for the Yorkshire Terrier breed and may highlight the variety in phenotypes of chronic enteropathies between different breeds.

Alterations in the metabolic profile reflecting disease activity would be helpful to monitor therapeutic responses in dogs with YTE. However, although we observed changes in the metabolite profile of dogs in clinical remission compared with untreated dogs, significance was lost after correction for multiple testing. We detected a decrease in 3-methionine-histidine without changes in 1-methionine-histidine, indicating improved muscle protein turnover in YTE after treatment [59]. This is consistent with improved muscle conditioning scores in our treated dogs. Furthermore, eicosadienoic acid showed a trend for recovery in YTE dogs after treatment, and a phosphatidylcholine seemed depleted in YTE dogs in remission compared to YTE dogs with active disease. Notably, changes in bile acid balance and acylcarnitines were not recovered after treatment. This might be the case if dysregulated metabolism is a predisposing factor for YTE or if the timeframe chosen for reassessment has been insufficient to revert metabolism alterations. In accordance with our results, in a previous study, clinical remission after a treatment period of 3 weeks with immunosuppressives and diet in IBD dogs was not accompanied by significant changes in the fecal microbiota or serum metabolite profiles [12]. However, in another study, treatment with a novel protein diet and treatment with prednisolone for 4 weeks had a significant impact on the phospholipid profile in dogs with chronic enteropathies [30]. Additionally, a recently published study revealed an altered lipid metabolism after 10 weeks of feeding a hydrolyzed diet to dogs with chronic enteropathies [60]. Therefore, the timeframe of 70 days chosen in our study should have been sufficient to reveal a recovery of altered metabolites.

We hypothesized that the plasma metabolite profile of dogs in clinical remission would change compared to dogs with active disease, resembling more closely the profile of healthy dogs. This was not the case. On the contrary, we found the highest numbers of metabolites with significant differences when comparing clinically silent YTE and healthy controls. In accordance with the differences between active YTE and healthy controls, dogs in clinical remission had changes in acylcarnitines, secondary bile acids, and long-chain fatty acids compared to healthy dogs. However, despite these alterations, YTE dogs in clinical remission showed elevated levels of several ceramides, phosphatidylcholines, sphingomyelins di- and triglycerides compared to healthy controls. At the same time, only a small minority of amino acids and amino acid-related compounds were different between the groups. These results are similar to a study evaluating the influence of feeding a hydrolyzed diet in dogs with IBD, in which phosphatidylcholines, sphingomyelins, di- and triglycerides showed the most significant increases in concentration after treatment [60]. Ceramides, sphingomyelins, and phosphatidylcholines are abundant in intestinal membranes, providing structural integrity and acting as receptors for the immune system [61,62,63]. The higher levels of phosphatidylcholines, sphingomyelins, and ceramides may reflect a high turnover rate of mucosal cells associated with restoring the mucosal barrier in treated dogs. After treatment, the increased di- and triglycerides may indicate improved lipid absorption by a recovered intestinal mucosa. In a recent study, feeding an omega-3 enriched diet increased the treatment response and resulted in marked suppression of intestinal inflammatory activity in IBD dogs [64]. However, diet may have an impact on plasma lipid profiles. Several studies have shown that diet can affect lipid metabolism [14,65], which could be a potential problem for the usefulness of lipid profiles as markers for disease control.

The main limitation of our study is the small sample size. Another limitation is that we did not perform gastroduodenoscopy in dogs in clinical remission. Since gastroduodenoscopy in dogs has to be performed under general anesthesia, it is not routinely used in the follow-up of canine IBD, and due to the relative invasiveness of these examinations, owners would not have given their consent. This is a limitation that will not easily be overcome in privately owned dogs. In the future, canine intestinal organoids from affected dogs may allow us to study underlying pathobiological mechanisms and evaluate the impact of different therapeutic strategies in vitro [66]. Furthermore, since we enrolled client-owned dogs, the dogs were not fed a standardized diet, which might have influenced metabolite profiling results. However, at the time of enrolment, all dogs except one (receiving a BARF diet) were fed comparable commercially available diets. Additionally, two dogs in the treatment group received prednisolone because they did not respond to the dietary treatment trial alone. This may have influenced our results in two ways. Prednisolone may have a direct effect on individual metabolites, and dietary nonresponders may represent a more severe disease phenotype. In the future, to ensure clinical utility, it might be necessary to include additional control groups, including patients with other gastrointestinal or inflammatory disorders.

In summary, our study identified changes in lipid and bile acid metabolism in Yorkshire Terriers with YTE. These changes might be breed-specific and might be involved in the pathogenesis of YTE. Notably, depletion of long-chain fatty acids and their acylcarnitines was observed in YTE patients. Considering the anti-inflammatory effects of fatty acids, further studies are needed to elicit the possible benefits of balancing metabolic lipid pathways to reduce inflammatory processes and improve treatment response in canine IBD. Recognizing and understanding breed-associated differences provides an excellent opportunity to optimize our treatment approach in canine IBD.

## 4. Materials and Methods

### 4.1. Cases and Control Dogs

Client-owned Yorkshire Terriers (*n* = 13) with YTE presented to the Small Animal Internal Medicine Clinic of the University of Veterinary Medicine Vienna, Austria, were prospectively enrolled between January 2019 and June 2021. Inclusion criteria were a history of chronic (≥3-week duration) or intermittent GI signs (vomiting, diarrhea, anorexia, or weight loss) or pleural or abdominal effusion. To qualify as YTE, a thorough diagnostic evaluation was performed to eliminate other possible causes for GI signs or effusions. Diagnostic tests included a physical exam, CBC, serum biochemical profile, measurement of bile acids and basal cortisol concentrations, ACTH-stimulation test (if basal cortisol < 2 µg/dl), and the assessment of serum concentration of cTLI (canine trypsin-like- immunoreactivity), SpecPL (specific pancreatic lipase), and cobalamin. Blood sampling included EDTA-plasma samples for metabolome analysis. Furthermore, urinalysis for urine protein creatinine ratio (UPC) and urinary sediment, abdominal ultrasonography, and analysis of fecal samples by flotation and fecal Giardia antigen test was performed. All YTE cases underwent gastroduodenoscopy at the time of presentation.

Adult Yorkshire Terriers without GI signs (*n* = 20) were prospectively enrolled as controls over the same time period. They were healthy as defined by history, physical exam, CBC, chemistry profile, fecal analysis, urinalysis, and abdominal ultrasound. Dogs with previous antibiotic or glucocorticoid treatment within the last 2 weeks and dogs with the previous feeding of a GI diet within the last 2 months were excluded.

Body conditioning scores (Nestle Purina: from 1-very thin to 9–significant obesity), fecal scores (Nestle Purina: form 1–very firm to 7–watery feces), and muscle conditioning scores (WSAVA global nutrition committee: from A–normal muscle mass to D-severe muscle loss) were recorded. Clinical scores were calculated for all dogs using the canine chronic enteropathy activity index (CCECAI), assessing the severity of alterations in 9 different categories, including attitude and activity, appetite, vomiting, fecal consistency, defecation frequency, weight loss, serum albumin concentration, peripheral edema and ascites, and pruritus [67]. Scores were recorded at the time of study enrolment and additionally for YTE dogs at re-evaluation appointments. One board-certified internist (A.I.G.) performed the initial diagnostic workup and the re-evaluations. One board-certified pathologist graded intestinal biopsies of the YTE dogs according to the World Small Animal Veterinary Association (WSAVA) International Gastrointestinal Standardization Group guidelines [2].

All dogs with YTE were part of a feeding trial and received the same treatment protocol. The cases were split up evenly into 2 treatment groups (random assignment with random number generation) and were fed either a hydrolyzed diet (Hill’s Prescription Diet z/d Canine) or a low-fat diet (Hill’s Prescription Diet i/d Low Fat Canine). The owners and the specialist performing the clinical re-evaluations were blinded regarding the diet. In both groups, the dogs were clinically re-evaluated after 14 days. If the treatment was unsuccessful (CCECAI score > 3), the dogs were switched to the other diet and re-evaluated after 28 days. A wash-out period was not considered due to ethical concerns. The feeding of canned or dry food was at the owner’s discretion to meet each patient’s individual needs. Dogs were fed the amount of food according to the manufacturer’s guidelines using their body weight. If dietary treatment alone was unsuccessful after 28 days (CCECAI score > 3), dogs received prednisolone (1 mg/kg q12h PO). If therapy was still unsuccessful after another 14 days, cyclosporine (5 mg/kg q24h PO) was added as a second immunosuppressive agent. After reaching clinical remission (defined by a decrease of CCECAI scores to ≤3), earliest 70 days after treatment initiation, YTE dogs were again re-evaluated, and blood work was repeated. Due to ethical reasons, gastroduodenoscopy was not repeated in dogs in clinical remission.

### 4.2. Sampling

For metabolome analysis, EDTA samples were taken from dogs with YTE at the time of diagnosis and after reaching clinical remission. Healthy control dogs were sampled once. EDTA tubes were centrifuged, and EDTA plasma was stored at −80 °C until analysis.

### 4.3. Metabolite Analysis

Plasma samples were analyzed using the MxP^®^ Quant 500 targeted metabolomics kit (Biocrates Life Sciences AG, Innsbruck, Austria). The kit provides coverage of up to 630 known metabolites and lipids from 26 biochemical classes and all relevant pathways. Compound classes covered are alkaloids (1), amine oxides (1), amino acids (20), amino acid-related metabolites (30), bile acids (14), biogenic amines (9), carbohydrates and related (1), carboxylic acids (7), cresols (1), fatty acids (12), hormones (4), indoles and derivatives (4), nucleobases and related (2), vitamins and cofactors (1), acylcarnitines (40), phosphatidylcholines (76), lysophosphatidylcholines (14), sphingomyelins (15), ceramides (28), dihydroceramide (8), hexosylceramides (19), dihexosylceramides (9), trihexosylceramides (6), cholesteryl esters (22), diglycerides (44) and triglycerides (242).

Sample preparation and analysis were performed according to the user manual provided by the manufacturer. In brief, 10 μL of samples were pipetted on a 96 well-plate, preloaded with internal standards to analyze the metabolite profile in the samples quantitatively. Next, a phenyl isothiocyanate (PITC) solution was added for derivatization of selected metabolites (e.g., amino acids); after the derivatization, the target analytes were extracted with an organic solvent, followed by a dilution step. The obtained extracts were then analyzed by FIA-MS/MS and LC-MS/MS methods using multiple reaction monitoring (MRM) to detect the analytes. Lipids and hexoses were measured by flow injection analysis-tandem mass spectrometry (FIA-MS/MS) using a Xevo TQ-S mass spectrometer (Waters, Vienna, Austria) with electrospray ionization (ESI) source, and other metabolites were measured by liquid chromatography-tandem mass spectrometry (LC-MS/MS) using a 5500 QTRAP^®^ mass spectrometer (AB Sciex, Darmstadt, Germany). For LC-MS/MS data analysis, concentrations were calculated using appropriate mass spectrometry software (Sciex Analyst^®^), and data were imported into Biocrates MetIDQ™ Oxygen software for further analysis. FIA-MS/MS data were processed using Biocrates MetIDQ™ Oxygen software. The obtained targeted metabolomics data are available in Table S1.

### 4.4. Statistics

Data processing, statistical analysis, and data visualization were performed using R (version 3.4.1 and 4.0), RStudio (version 1.4.1717), and Metaboanalyst version 5.0.

#### 4.4.1. Data Cleaning and Imputation

The threshold was set to allow a maximum of 20% missing or below limit of detection (LOD) values. If at least 80% of valid values above LOD per analyte were available in at least one group, the analyte was included for further statistical analysis; otherwise, it was excluded. Values below LOD were replaced by values between LOD and LOD/2 using a logspline imputation method developed for data being right-censored, left-censored, or interval-censored [68]. 522 metabolites remained after data cleaning and imputation. Further data log 10-transformation of the cleaned and imputed data set was performed.

#### 4.4.2. Univariate and Multivariate Statistics

A fixed linear model ANOVA performed statistical analysis and comparison between groups. Dogs before treatment and in clinical remission were analyzed in a separate mixed linear model ANOVA with the group as fixed and dog ID as a random effect. *p* values < 0.05 were considered significance. For correction of the false recovery rate in multiple comparisons, q-values were calculated using the Benjamini and Hochberg method [69]; a q-value of < 0.2 was considered statistically significant. Multivariate data analysis included principal component analysis (PCA) and partial least-squares-discriminant analysis (PLS-DA).

## Data Availability

The targeted metabolomics data presented in this study are available in Supplementary Materials Table S1.

## Supplementary Materials

The following supporting information can be downloaded at: https://www.mdpi.com/article/10.3390/metabo12030264/s1, Table S1: Targeted metabolomics data.
